# Arterial Thrombosis of Unknown Etiology in a 10-Year-Old Male

**DOI:** 10.7759/cureus.25400

**Published:** 2022-05-27

**Authors:** Dhiaeddine Djabri, Azd Al-Mashal, Darshan Rola, Andrew Galligan

**Affiliations:** 1 Pediatrics, University of South Florida Morsani College of Medicine, Tampa, USA; 2 Pediatrics, Nova Southeastern University Dr. Kiran C. Patel College of Osteopathic Medicine, Fort Lauderdale, USA; 3 Pediatric Hematology and Oncology, University of South Florida Morsani College of Medicine, Tampa, USA

**Keywords:** anticoagulation, elevated factor viii, aspirin therapy, von willebrand factor, thrombophilia, genetic risk factors, pediatric thrombus, arterial thrombosis

## Abstract

Arterial thrombotic events are exceedingly rare occurrences in pediatric populations. The incidence of childhood thrombosis is extremely low and the majority of cases are venous in origin. There are several well-known etiologies and genetic risk factors with an underlying predisposition to venous thrombosis, however, there have been few identified etiologies of arterial thrombotic events in pediatric patients. The most common include factor V Leiden mutation, trauma, neoplasm, and inherited metabolic disorders. This case report involves a 10-year-old male with no predisposing factors or significant medical or surgical history, who presents with a cerebral vascular accident secondary to a peripheral arterial clot of the basilar artery. The patient's only identifiable risk factor was an elevated factor VIII level. Elevated factor VIII levels are a risk factor for thrombotic events, with a greater impact on venous than on arterial thrombosis. However, due to a lack of international consensus on methods for the laboratory testing of factor VIII levels in plasma, it is not currently recommended that the measurement of factor VIII levels be part of routine thrombophilia screening in pediatric populations.

## Introduction

Though the incidence of pediatric stroke is low in comparison to the adult population, it remains an important cause of chronic morbidity and mortality in children and can lead to a multitude of disabilities including motor, cognitive, and behavioral dysfunction. Within the age range of 0 days to 18 years, population-based studies estimate an annual incidence rate of acute ischemic stroke of 4.6 per 100,000 persons [1]. Traditionally, the risk factors for childhood acute ischemic stroke have included congenital cardiovascular disease, sickle cell disease, infection, and hypercoagulable states. Regarding cerebral ischemic events, thrombosis in the central nervous system may occur in either the sinovenous system (more common) or the arterial system (less common). Predominantly, clot formation in the venous system results from sites of reduced flow, whereas clots in the arterial system involve platelet aggregation at high shear rates. An international Pediatric Stroke Study reported an incidence of 13% of acute ischemic stroke to be due to a hypercoagulable state, making it the third leading cause of acute ischemic stroke after cardiovascular disease and infection [2]. These hypercoagulable states include systemic lupus erythematosus, prothrombin G20210A mutation, homocystinuria, factor V Leiden mutation, and deficiencies in antithrombin, protein C and S, and plasminogen. Even though some of these phenotypes are usually associated with venous hypercoagulation, some have also been shown to cause arterial thrombosis. More specifically, recent data has shown that multiple laboratory phenotypes have also been associated with venous thrombosis, including an increased level of factor VIII [3]. Factor VIII has also been associated with an increased risk of arterial thrombosis in coronary heart disease and stroke [4]. Therefore, detecting acquired and congenital prothrombotic disorders is important because this information remains a highly debated topic for the long-term management of the pediatric population. 

Primarily, this case report portrays the case of a previously healthy 10-year-old male patient with an elevated factor VIII level, who developed a basilar artery acute ischemic infarction. Currently, the literature available describes a diagnosis of arterial thrombosis with elevated factor VIII in only a few publications in adults [5], making this report a rare documentation of acute arterial cerebral infarction (with elevated factor VIII) in the pediatric population.

## Case presentation

The patient is a 10-year-old male with no significant past medical history who presented to the emergency department (ED) for altered mental status. Upon waking up in the morning, the patient experienced several episodes of vomiting and experienced some dizziness. As the morning progressed, he complained of worsening dizziness, new-onset blurry vision, and right-sided weakness. The patient was transported to ED and on arrival, was unable to speak, was experiencing right upper extremity jerking motions, and right eye deviation. Once in the ED, MRI and MR angiography of the brain were obtained, demonstrating acute left pontine stroke secondary to a basilar artery occlusion. The patient subsequently underwent a successful mechanical thrombectomy. The thrombophilic workup (Table 1) was negative apart from an elevated factor VIII level at 235.8% (normal: 50% to 150%) which continued to increase to 305% on follow-up labs. A positive parainfluenza 2 viral PCR was obtained as well. Complete blood count was normal and there were no signs of a myeloproliferative disorder. A transesophageal echocardiogram bubble study was performed and it showed no signs of a patent foramen ovale or other cardiac defects. Metabolic testing showed normal acylcarnitine and homocysteine levels. Genetic testing yielded no evidence of the prothrombin G20210A gene, factor V Leiden gene, or MTHFR gene mutations. Infectious workup was positive for parainfluenza 2 but otherwise negative for other respiratory viral PCR panels including SARS-2 Covid and Herpes Simplex 1 & 2. It was initially unclear as to the cause of the elevation in factor VIII, as factor VIII is an acute phase reactant and may remain elevated following an acute illness or following a surgical procedure. The patient was placed on daily aspirin 325mg therapy, with plans to follow up serial factor VIII levels to determine the exact etiology. Finally, the patient had multiple EEGs for seizure monitoring during hospital admission as well as levetiracetam for seizure prophylaxis.

**Table 1:** Post-admission hypercoagulation workup. PCR: polymerase chain reaction; RNA: ribonucleic acid; MRSA:  methicillin-resistant Staphylococcus Aureus; CPK: creatine phosphokinase; UMOL/L: micromole per liter; MG/DL: milligram per deciliter; U/mL: units per milliliter; U/L: units per liter

Respiratory viral PCR Panel		
Component	Results	
Adenovirus PCR	Not detected	
Coronavirus 229E PCR	Not detected	
Coronavirus HKU1 PCR	Not detected	
Coronavirus NL63 PCR	Not detected	
Coronavirus OC43 PCR	Not detected	
Human metapneumovirus PCR results	Not detected	
Rhino/enterovirus PCR	Not detected	
Influenza A PCR	Not detected	
Influenza B PCR	Not detected	
Parainfluenza 1	Not detected	
Parainfluenza 2	Detected	
Parainfluenza 3	Not detected	
Parainfluenza 4	Not detected	
Respiratory syncytial virus RNA	Not detected	
Bordetella pertussis PCR	Not detected	
Chlamydophila pneumoniae	Not detected	
Mycoplasma pneumoniae	Not detected	
SARS-COV-2 AB IgG, serum/plasma	Negative	
Coagulation molecular workup		
Component	Value	Reference Range & Units
Antithrombin III (3)	80	83 - 128 %
Protein C activity	58	70 - 140 %
Protein S activity	85	64 - 149 %
Factor 5 mutation	Negative for the presence of the factor V gene R506Q mutation.	
Factor 2 mutation	No prothrombin (Factor II) G20210A gene variant detected.	
4 H folate mutation	Positive for one copy of the C677T variant and one copy of the A1298C variant	
Homocysteine	1.7 UMol/L	5 - 20 UMol/L
Fibrinogen level	285 mg/dL	190 - 500 mg/dL
Cardiolipin AB IgG	<1.6 U/mL	Negative : < 20 U/mL Positive : >20 U/mL
Cardiolipin AB IgM	<0.2 U/mL	Negative : < 20 U/mL Positive : > 20 U/mL
Cardiolipin AB IgA	<0.5 U/mL	Negative : < 20 U/ml Positive : > 20 U/mL
Antinuclear antibody	Negative	
Other medication/substances/infections		
Amphetamine	Negative	
Benzodiazepines	Negative	
Cannabinoids	Negative	
Cocaine	Negative	
Opiates	Negative	
Herpes simplex PCR 1	Not detected	
Herpes simplex PCR 2	Not detected	
MRSA	Not detected	
CPK	363 U/L	30 - 200 U/L

## Discussion

When searching for the causes of stroke, it is worth examining the coagulation system, including factor VIII concentration, for its moderate association with venous thromboembolism, coronary artery disease, and stroke [6-8]. Currently, there are limited cases associating factor VIII and acute ischemic stroke in the adult and pediatric population.

However, the literature does have some indirect supportive evidence indicating a heritable correlation between elevated factor VIII and families with thromboembolic events. Kamphusien et al. reported a five to six-fold increase of venous thrombotic events in families with a high factor VIII level (>150 IU/dl), as well as a genetic clustering within affected families [9]. Additional supporting evidence for the genetic link of factor VIII and ischemic stroke came from a retrospective study of 177 patients, in which 40% of adult patients with a history of arterial thrombosis had a first-degree relative with elevated factor VIII [10].

In addition, after adjusting the blood group, vWF level, and age, a single gene polymorphism in the ABO locus (rs505922) showed that elevated factor VIII levels remain an independent risk factor for ischemic stroke. In relation to myocardial infarction, the Caerphilly Heart Study, and The Atherosclerosis Risk in Communities Study both reported an increased risk of ischemic heart disease in patients with elevated factor VIII levels [11,12]. Furthermore, Lasek-Bal et al. found increased activity of factor VIII in five out of nine adult patients, eight to 10 months following a cerebral stroke episode [13].

Genetic abnormalities are always considered on the differential of arterial thrombi, especially in pediatric populations. Several established genetic and acquired conditions are known to compromise hemostasis or coagulation and predispose to premature thromboembolic events. Due to the lack of research and presentation of patients with excess factor VIII resulting in arterial thrombi, there is no established therapeutic intervention. Based on the patient's past medical history, past surgical history, laboratory work up, and genetic testing it was concluded that high factor VIII levels were most likely the cause of his acute thrombotic event. Elevated factor VIII levels have been identified as an independent risk factor for thrombosis, although they tend to be more associated with venous thrombosis, rather than arterial thrombosis.

The question that remains is whether the factor VIII elevation was the cause of the acute thrombotic event or if it was simply elevated secondary to an acute presentation, surgical procedure, or other reasons. The patient was followed by pediatric hematology/oncology hematology and underwent serial factor VIII monitoring for the next six months.

When considering the etiology of the increased factor VIII levels, consideration of trauma vs genetic factors vs exogenous causes was considered. Trauma remains a potential cause of the elevations in factor VIII level, given the mechanical thrombectomy that the patient underwent to remove the basilar artery occlusion. Factor VIII is stored in the endothelial lining of vessels, with catheter-associated advanced procedures having have been known to acutely elevated factor VIII levels post-op. Exogenous causes that would place the patient in a hypercoagulable state were also considered including potential drug causes, and exogenous hormone sources. Drug screening on admission was negative, and hormone levels were within the reference range for his developmental age group. The only other positive finding in the patient's lab work was a positive titer for parainfluenza 2. Infections can be associated with a hypercoagulable state and this could have contributed to the patients presentation but there is limited data to support this link. Though there are other factors associated with elevated factor VIII levels, they remain poorly described. However, this provides a potential area for of further research, as increasingly sophisticated sequencing and molecular studies are available for screening of potential atherothrombotic factors.

Therapeutic options for long term anticoagulation therapy were limited given the patient’s age. A previous case study examining an adult with a peripheral venous thrombosis secondary to factor VIII elevation recommended oral anticoagulation therapy [14]. Given our patient’s age and response to daily aspirin, the patient was placed on a six-month trial of aspirin therapy, pending follow up lab work.

## Conclusions

This case involves a unique presentation of a 10-year-old male with an arterial thrombus. A complete workup was done to identify the cause and the only abnormality was an elevated factor VIII level. There are minimal reported cases involving an arterial thrombus formation secondary to elevated factor VIII, and this case aims to possibly shed light on a relatively rare cause of a hypercoagulable state in a pediatric patient. It is reasonable to assume the elevated levels cause the arterial thrombus based on the workup, however, the complete molecular basis for elevated factor VIII remains unclear. Due to the extremely low incidence of factor VIII elevation in the adult and pediatric populations, it is generally not included in routine thrombophilia screening. Further investigation is needed before it is incorporated into this screening. Both genetic and environmental elements seem to affect it including age, smoking, exercise, stress, and surgery, among many others. This can lead to significant fluctuations in its levels thus creating a need for appropriate timing of the measurement. There are also no established values or ranges, in which the levels of factor VIII must exceed, in order for thrombotic events to become more likely. These are important issues that must be answered before testing of factor VIII has any diagnostic or prognostic value. Currently, there are no specific treatments aimed to specifically combat elevated factor VIII levels. This poses another challenge that must be solved. The typical anticoagulant regimen may be effective in preventing thrombus formation in patients with elevated factor VIII, but long-term studies are needed in order to ensure their efficacy. Overall, further studies and trials are needed before a screening and treatment regimen is created to combat the hypercoagulable state caused by elevated factor VIII.
